# Efficacy of repetitive transcranial magnetic stimulation on chronic migraine: A meta-analysis

**DOI:** 10.3389/fneur.2022.1050090

**Published:** 2022-11-24

**Authors:** Jiugen Zhong, Wanting Lan, Yanqing Feng, Ligen Yu, Rang Xiao, Yingying Shen, Zhi Zou, Xiaohui Hou

**Affiliations:** ^1^College of Kinesiology, Shanghai University of Sport, Shanghai, China; ^2^School of Sport and Health, Guangzhou Sport University, Guangzhou, China

**Keywords:** repetitive transcranial magnetic stimulation, rTMS, chronic migraine, efficacy, meta-analysis

## Abstract

**Introduction:**

Migraine is a neurovascular disorder that affects the quality of life of more than 1 billion people worldwide. Repetitive transcranial magnetic stimulation (rTMS) is a neuromodulation tool that uses pulsed magnetic fields to modulate the cerebral cortex. This meta-analysis ascertained the therapeutic or preventive effect of rTMS on chronic migraine.

**Methods:**

We performed a database search of PubMed, Web of Science, Embase, and the Cochrane Library from January 2004 to December 2021. Eligible studies included randomized controlled studies of the analgesic effects of rTMS in patients with chronic migraine.

**Results:**

Eight studies were included. Random effects analysis showed an effect size of −1.13 [95% confidence interval (CI): −1.69 to −0.58] on the frequency of migraine attacks, indicating that rTMS was more effective for decreasing migraine attacks than the sham rTMS.

**Conclusions:**

The meta-analysis revealed that rTMS is an effective approach for reducing migraine attack when the dorsolateral prefrontal cortex was stimulated. However, rTMS may not be suggested as a method to reduce the pain level.

**Systematic review registration:**

http://www.crd.york.ac.uk/PROSPERO/, identifier: CRD42021228344.

## Introduction

Migraine is a neurovascular disorder that affects more than 1 billion people worldwide. Its widespread prevalence and associated disabilities have a range of negative and substantial impacts not only on directly affected patients but also their families, colleagues, employers, and society (1), as well as a high medical burden (2). Despite its widespread prevalence, migraine remains under- diagnosed and under-treated (3). In general, to eliminate the exacerbating factors, some interventions were be used, for example, lifestyle management (4, 5) and pharmacological treatment (6, 7). Of note, pharmacological treatment has been shown to be effective for migraine, but there are still large individual differences (3), and may bring side effects such as a rapid and progressive headache worsening following anti-CGRP monoclonal antibodies treatment suspension (8). A pilot randomized trial found that both botulinum toxin-A (BTX-A) and repetitive transcranial magnetic stimulation (rTMS) were well tolerated and effectively for chronic migraine prophylaxis (9), however, the side effects of BTX-A need to be carefully considered. Therefore, non-pharmacological treatments could serve as a safer and effective method for the management of migraine are needed.

Non-invasive brain stimulation (NiBS) technology has been regarded as an important innovation in neuropsychiatric diseases and chronic pain (including migraine) in recent years and widely used in clinical settings (10–13). Transcranial magnetic stimulation (TMS), which applied a magnetic field to the surface of the scalp and induces current in the subjacent cortex (14), is an effective and safe approach that has been approved by the FDA for migraine treatment (15, 16). As a NiBS method, TMS can excite or depolarize neurons by a fast alternate magnetic field (17), and electrical changes in the brain are believed to regulate neurotransmitters in the brain (14). TMS may reduce pain by modulating the excitability level (14), as patients with migraine tend to show hyperexcitability of the neurons (18). rTMS is a type of TMS, which can deliver a repeated series of magnetic impulses to the cortex (14). Compared to single or paired-pulse TMS, rTMS shows increasing significance as the plastic effect lasts long after the stimulation (19, 20). Chronic migraine patients may also suffer a higher level of central excitability (21). So, the long-lasting effect of rTMS could be appropriate for chronic migraine sufferers.

Several meta-analyses have demonstrated the effect of TMS, rTMS, and tDCS (22–24) on headache, but no review has focused on the effect of rTMS on chronic migraine. As the main effect of rTMS is to modulate the activation level of the cortex, most of the previous evidence showed that rTMS could be mainly used for migraine prophylaxis (25). However, growing evidence has demonstrated the treatment effect of rTMS on migraine in recent years. For instance, Fierro et al. (26) demonstrated that high frequency TMS stimulation on the motor cortex could significantly decrease the pain level of patients with chronic migraine, while the efficacy of the treatment of rTMS on migraine is still under debate. One reason for the uncertain treatment effect could be the different stimulation site. Some studies have shown that stimulation in the motor cortex could reduce pain (27, 28), while others have demonstrated that stimulation on the left dorsal prefrontal cortex (LDLPFC) could decrease the frequency of headache attacks (29, 30). However, no evidence has demonstrated the effect of stimulating different sites on patients with chronic migraine.

Therefore, this meta-analysis explored the effect of rTMS on chronic migraine with or without aura. We first analyzed the treatment and prophylaxis effect of rTMS on chronic migraine indexed by the pain intensity and frequency of headache attacks, respectively. The relationship between the stimulation site and efficacy of rTMS was also analyzed.

## Methods

The protocol was registered in the International Prospective Register of Systematic Reviews (https://www.crd.york.ac.uk/PROSPERO) with registration number CRD42021228344.

### Literature search

A literature search was conducted for studies published in the past 20 years up to December 25, 2021, of studies indexed in four electronic databases, including PubMed, Embase, Web of Science, and the Cochrane Library. The keywords used for identifying rTMS were “repetitive transcranial magnetic stimulation” and “rTMS,” while the keywords used for identifying migraine were “migraine disorder” and “migraine^*^.” The language was restricted to English. The detail of searching strategies was provided in Supplementary material.

### Inclusion criteria

First, articles from the electronic database were initially screened by title and abstract. Two reviewers (Z.J.G and Z.Z) independently screened the title and abstracts of studies to determine whether they met the selection criteria (Table 1). Any disagreement was solved by consensus or by discussion with the third reviewer (H.X.H). Finally, the full texts were analyzed. The detail inclusion criteria were follows: (1) Human study; (2) Parallel or crossover RCT design; (3) Patients with chronic migraine (with/without aura), diagnosed according the International Classification of Headache Disorders (ICHD, 2nd edition) (31); (4) Types of intervention, including rTMS intervention by single or multiple stimulation; (5) Main outcome indicated that pain level was assessed on a visual analog scale (VAS) or numerical pain rating scale (NPR),and the row data can be extracted from tables or figures. However, the study was excluded that: (1) Did not meet the inclusion criteria; (2) Published without peer review; (3) Treatment paradigm was outside the published safety guidelines.

**Table 1:** Eligibility criteria for considering articles for the review.

	**Inclusion**	**Exclusion**
Participants	Chronic migraine patients with and/or without aura, age >15 years	Migraine patients with medication overuse or headache after trauma
Intervention	Studies that applied rTMS as a prevention or intervention method	Presented results of rTMS associated with other interventions
Comparison	The control group only received a placebo (e g., sham) or waiting list	
Outcome	Pain intensity measured by VAS or NRPS, frequency of migraine attacks reported by days/month	
Trial design	Randomized controlled clinical trials	Non-controlled studies
Type of publication	Original article and published in a peer-reviewed journal; language is English	

rTMS, repetitive transcranial magnetic stimulation; NPRS, Numeric Pain Rating Scale; VAS, Visual Analog Scale.

### Bias risk assessment

The quality of the included studies was examined by S.Y.Y and F.Y.Q using the bias risk assessment standards of the Revman 5.3.5 software. Two levels of low and high risk were used for evaluation. If the method used in this complied with the standard of assessment checklist, the risk was considered low; otherwise, if the method did not comply with that of assessment checklist, the risk was considered high. If no corresponding basis was found in the original text or if it was not reported, it was rated as “unclear.”

### Outcome measurement

We considered the outcome measures performed at the end of the follow-up. The primary outcome focused on pain intensity evaluated by VAS or NPRS and frequency of headache attacks (days/month). The VAS and NPRS scale will be uniformly converted to a rating scale of 1–10 if the rating scale is 1–100. To reduce the heterogeneity of the research as much as possible, only the post-treatment results at the 1-month (or 4-week) follow-up were extracted, which was used in most studies, when the research had multiple follow-up time nodes.

### Data analysis

Revman 5.3.5 software, which was developed by the Cochrane Collaboration, was used for statistical analyses. This analysis was performed separately by two authors (L.W.T and Y.L.G). Data extraction mainly comprised the sample size (for experiment and control groups), sex, age, area of stimulation, parameters of rTMS application (frequency, duration, interval, pulses times), pain intensity, and frequency of headache attacks (the baseline and following up time). The difference in mean value was calculated by Mean^post^ – Mean^pre^, and for the difference in the standard deviation, we used the following formula: SD = √ (SD^pre^^∧^2 + SD^post∧^2 – 2^*^0.04^*^ SD^pre^^*^SD^post^). The random-effects model was applied and statistically significant heterogeneity was assumed when the *P* value was < 0.05. The quantity I^2^ described the degree of heterogeneity with values of 25, 50, and 75% considered low, moderate, and high, respectively. To explore the possible cause of heterogeneity among study results, the subgroup analysis was used.

## Results

### Inclusion and selection of studies for meta-analysis

**Figure 1:**
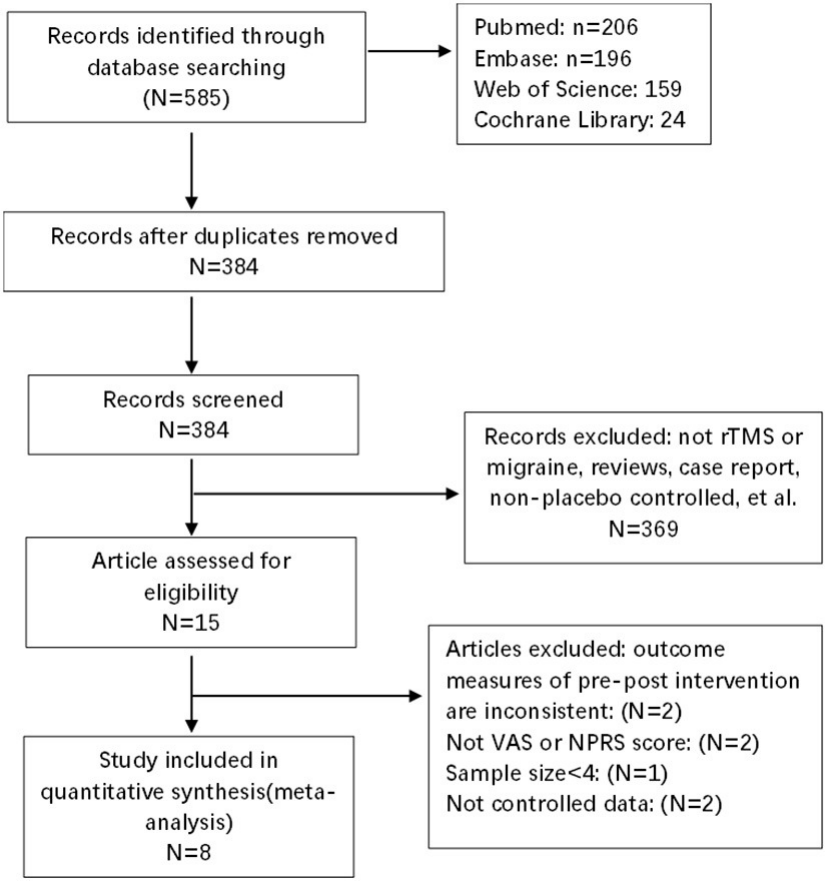
PRISMA flow diagram.

The search strategies yielded 585 results. After the removal of duplicates, 384 articles were identified, after reading titles and abstracts, case-reports and articles that had non-randomized sham-controlled designs, incomplete outcomes, and small sample sizes (*n* < 4) were excluded. Of these, eight were included in the quantitative analysis, with 199 migraine patients and 180 control patients. The details of the study selection are shown in Figure 1. The characteristics of the demographics of the subjects are shown in Table 2. The included studies were published between January 01, 2004 and December 25, 2021. The parameters of rTMS application and the main outcomes of each study are shown in Table 3.

**Table 2:** Demographic characteristics of subjects.

**References**	**Population**	**Sample size (E/C)**	**Gender (f/m)**	**Age (year, Mean ±SD): E/C**
Misra et al. (32)	Chronic migraine with/without aura	71 (24/47)	56/15	35.0 ± 11.40/34.1 ± 9.43
Amin et al. (29)	Chronic Migraine with/without aura	33 (14/19)	28/5	37.4 ± 11.7/ 32.2 ± 9.8
Kumar et al. (30)	Chronic migraine without aura	20 (10/10)	11/9	33.2 ± 8.2/ 33.80 ± 7.2
Todorov et al. (27)	Chronic migraine without background headache	66 (38/28)	53/13	40.2 ± 11.05/36.9 ± 10.28
Todorov et al. (27)	Chronic migraine without background headache	65 (37/28)	52/13	38.7 ± 11.05/36.9 ± 10.28
Brighina et al. (25)	Chronic migraine (meet IHS 2nd edition)	11 (6/5)	7/4	47.0 ± 7.0
Misra et al. (33)	Chronic Migraine with/without aura	100 (50/50)	88/12	35.6 ± 10.07/ 35.1 ± 10.38
Teepker et al. (28)	Chronic Migraine with/without aura	27 (14/13)	22/5	30.7 ± 8.94/40.62 ± 11.53
Sahu et al. (34)	Chronic Migraine with/without aura	41 (20/21)	31/10	31.4 ± 7.51/30.23 ± 9.02

E, experiment group; C, control group; f, female; m, male.

**Table 3:** Characterize of rTMS application.

**Study**	**Area of stimulation**	**Parameters (including follow up time)**	**Main outcome (mean** ±**SD, E/C)**		**Sham stimulation parameters**	**Side effect**
			**Pain intensity(pre/post)**	**Attacks frequency (pre/post)**		
Misra et al. (32)	Left motor cortex	10 Hz, 600 pulses in 10 trains, 3 sessions (**1 month**)	No post VAS or NRPS	E: 22.80 ± 9.20/17.17 ± 8.43	A figure of 8 coil of 7 cm, similar sound without delivering any stimulus	Not reported
				C: 16.6 ± 10.8/9.47 ± 9.63		
Amin et al. (29)	LDLPFC	5 Hz, 900 pulses/session over 3 min duration, 5 sessions, 1week (**1 month**)	E: (NRPS)8.5 ± 1.5/ 6.4 ± 1.8	E: 9.3 ± 1.9/5.5 ± 3.2	A figure-of-eight (MC-B70) coil, 5-Hz, 50% motor threshold intensity, perpendicular to the brain surface over the left DLPFC site	Not reported
			C: (NRPS)9.1 ± 1.2/7.7 ± 1.9	C: 7.3 ± 2.9/6.1 ± 2.7		
Kumar et al. (30)	M1	10 Hz, 60s interval, 60 pulses/trains, 10 min/session, 5 days/week, 2 weeks (**1 month**)	E: (VAS)8 ± 1.33/4.2 ± 2.04 C: (VAS)7.7 ± 1.42/4.8 ± 2.25	E: 17.40 ± 1.33/10.2 ± 2.21 C: 17.6 ± 1.42/18 ± 1.6	Perpendicular to the vertex at the minimum stimulation strength of the stimulator, similar sound without delivering any stimulus	Not reported
Todorov et al. (27)	M1	15 Hz, 10s intertrain interval, 30 pulses/train, 40 trains. (**1 month**, 2 month)	E: (VAS)9.0 ± 0.93/6.9 ± 2.55 C: (VAS)9.1 ± 1.33/7.9 ± 2.88	E: 14.5 ± 4.49/7.7 ± 6.97 C: 14.1 ± 6.33/13.2 ± 7.02	A figure of eight coil, same parameters, perpendicular to the surface of the scalp	No serious adverse events
Todorov et al. (27)	LDLPFC		E: (VAS)9.3 ± 0.93/6.9 ± 2.55	E: 13.8 ± 5.42/7.8 ± 5.39		
			C: (VAS)9.1 ± 1.33/7.9 ± 2.88	C: 14.1 ± 6.33/13.2 ± 7.02		
Brighina et al. (25)	LDLPFC	20 Hz, 10 trains of 2s duration, 30s intertrain interval, 12 session (**1 month**, 2 month)	Headache index	E: 24.8 ± 2.6/11.8 ± 6.4 C: 24.6 ± 2.15/22.8 ± 1.83	A water cooled figure-of-eight coil, perpendicular to the brain surface over the left DLPFC site	Not reported
Misra et al. (33)	Left frontal cortex	10 Hz, 600 pulses in 412.4 s, 10 trains, 45 s intertrain interval (**1 month**)	No post VAS or NRPS	E: 20.8 ± 9.5/5.2 ± 4.9 C: 17.04 ± 10.30/8.9 ± 6.6	An air-cooled figure- eight coil of 7 cm diameter, producing similar sounds without giving magnetic pulses	1 drowsiness
Teepker et al. (28)	Vertex	1Hz, 2 trains of 500 monophasic pulses, 1 min intertrain interval, 5 days. (**8week**)	E: (NRPS)6.26 ± 1.33/6.11 ± 1.26 C: (NRPS)5.52 ± 1.72/5.17 ± 2.51	Not reported (only figure showed)	A figure of eight coil, producing the same sound and similar sensory feedback without delivering active stimulation	Sleepiness (Placebo Verum = 1:1), Headache (2:0), Amyostasia (1:1), Testiness (1:0), Vigorous dreams (0:1), Phonophobia (0:1), Drop-outs (1:1)
Sahu et al. (34)	LDLPFC	5 Hz, last for 2 s per train, 8 s interval, 20 train, 600 pulses per session (2 week, 4 week, 6 week)	E: (NRPS)7.15 ± 0.77/5.40 ± 1.10 C: (NRPS)6.58 ± 0.90/6.27 ± 0.88	E: 30 ± 7.8/15.3 ± 7.2 C: 25.2 ± 5.1/24.6 ± 6.9	A figure-of-eight-shaped coil, perpendicular to the brain surface over the left DLPFC site, same parameters	No serious adverse effects

M1, Primary Motor Cortex; LDLPFC, Left Dorsolateral Prefrontal Cortex; NPRS, Numeric Pain Rating Scale; VAS, Visual Analog Scale; E, Experiment group; C, Control group. The bold values represent the follow-up time when the main results of this study were extracted.

### Quality of the literature

**Figure 2:**
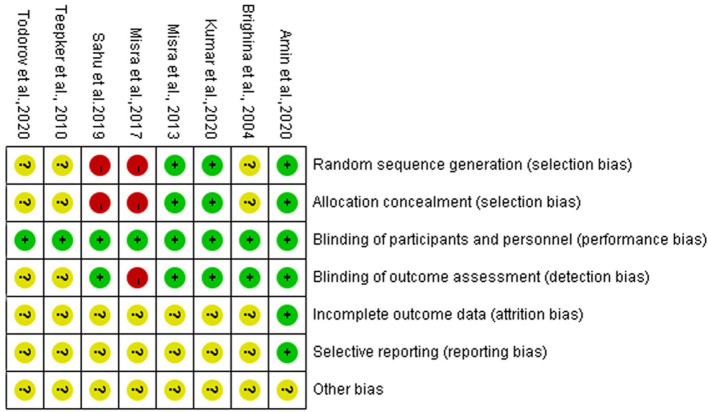
Quantity of the literature.

As shown in Figure 2, in eight studies, only one study by Amin et al. (29) reported the clinical identifier, which was considered a rigorous RCT study. Two articles (30, 33) detailed the random assignment method and were double-blinded (subject and evaluator blind), and four articles used randomization but either did not elaborate on the specific method (25, 27, 28) or the random method was inappropriate (34). One article used a placebo control but did not report whether it was randomized (32). Only one study was high quality, two were medium quality, and the remaining five were low-quality studies.

### rTMS protocols

Of the included studies, the parameters of rTMS application were heterogeneous. First, for the area of stimulation, three out of eight studies were stimulated at the left dorsolateral prefrontal cortex (LDLPFC), and four out of eight were stimulated at the primary motor cortex (M1) or vertex. One study by Todorov et al. selected both the LDLPFC and M1 as locations of stimulation. Second, the frequency use in four of eight papers was 10 Hz (30, 32, 33), 5 Hz was applied by Amin et al. (29) and Sahu et al. (34), 15 Hz was applied by Todorov et al. (27), 1 Hz was applied by Teepker et al. (28), and 20 Hz was applied by Brighina et al. (25); a 600–1,200 pulse was applied in these studies. Third, the duration of treatment were 5 sessions delivered in consecutive days in most studies (27–29, 34), 3 sessions and 12 sessions were delivered on alternate days by Misra et al. (32, 33) and Brighina et al. (25), respectively. Ten sessions were delivered on consecutive days in the study by Kumar et al. (30). The sham rTMS protocols is similar to real rTMS.

### Long-term analgesic effects of rTMS on migraine

**Figure 3:**
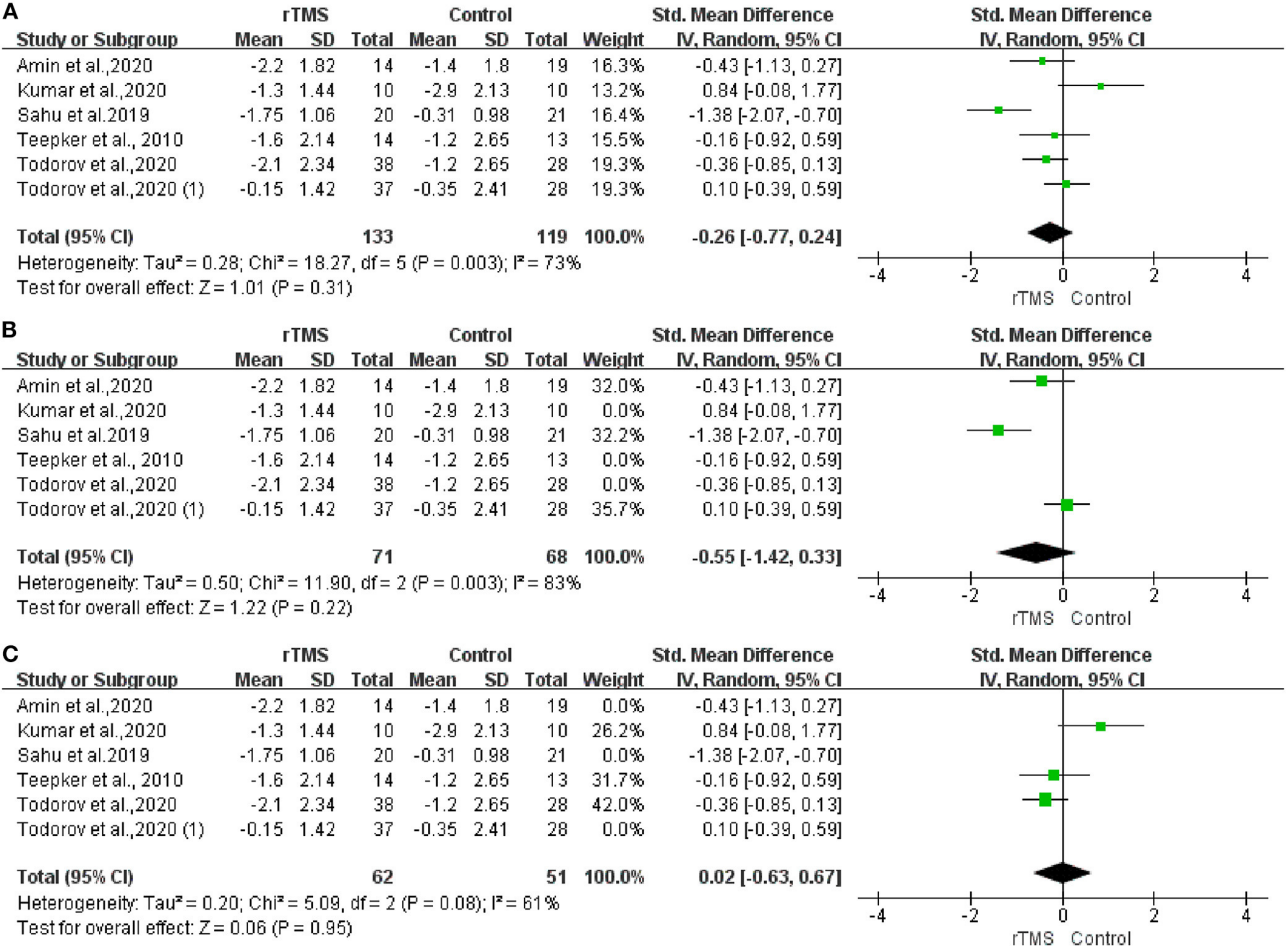
Forest plot of pain intensity. **(A)** LDLPFC/motor cortex stimulation. **(B)** LDLPFC stimulation. **(C)** Motor cortex stimulation.

To quantify the rTMS effects on migraine intensity, we performed an overall meta-analysis considering both the LDLPFC and M1 stimulation location. The results showed no significant difference between the real and sham rTMS groups in either LDLPFC or M1 region stimulation. However, moderate heterogeneity existed [I^2^ = 73%; *P* = 0.31; SMD: −0.26; 95% confidence interval (CI): −0.77 to 0.24, Figure 3A]. Additionally, no significant difference was observed after we performed a subgroup analysis on different stimulation locations for LDLPFC (I^2^: 83%; *P* = 0.22; SMD: −0.55; 95% CI: −1.42 to 0.33, Figure 3B) nor M1 (I^2^: 61%; *P* = 0.95; SMD: 0.02; 95%CI: −0.63 to 0.67, Figure 3C).

### Prevention effects of rTMS on migraine re-attacks

**Figure 4:**
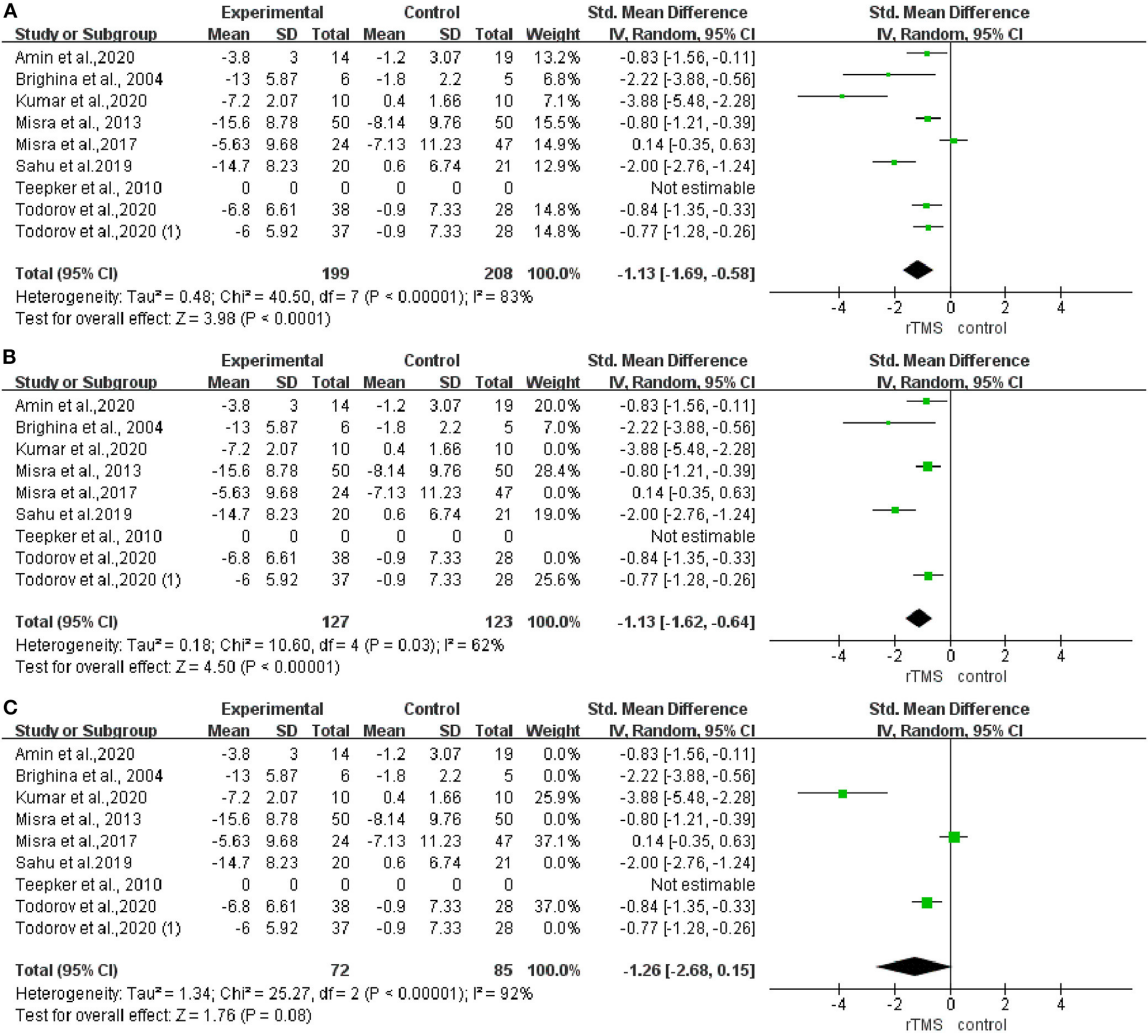
Forest plot of the frequency of headache attacks. **(A)** LDLPFC/motor cortex stimulation. **(B)** LDLPFC stimulation. **(C)** Motor cortex stimulation.

After analyzing the rTMS effects on the frequency of migraine attacks, the pooled standardized mean difference (SMD) effect showed that real rTMS was significantly more effective for decreasing migraine attacks than the sham rTMS, with a high heterogeneity (I^2^ = 83%; *P* < 0.001; SMD: −1.13; 95%CI: −1.69 to −0.58, Figure 4A). Meanwhile, the results of subgroup analysis showed that the rTMS decreased migraine attack frequency when the stimulation was applied to the LDLPFC (I^2^ = 62%; *P* = 0.03; SMD: −0.13; 95%CI: −1.62 to −0.64, Figure 4B). However, there was no effect when the stimulation was applied to the M1 cortex (I^2^ = 92%; *P* < 0.001; SMD: −1.26; 95%CI: −2.68 to 0.15, Figure 4C).

### rTMS adverse effects

Of eight studies, four reported no adverse effects (29, 30, 32, 35), three reported some adverse effects, such as sleepiness and mild dizziness (28, 33, 34), and only one study reported headache attack during treatment, the stimulation located on the M1 (27). No serious adverse effects were reported.

## Discussion

NiBS technology has been regarded as an important innovation in neuropsychiatric diseases and chronic pain including migraine (13). This meta-analysis aimed to explore the effect of rTMS on chronic migraine in different stimulation sites and evaluate the efficacy in terms of pain intensity and headache frequency. Consistent with previous results, we revealed that rTMS is an effective method in migraine prevention and that both the LDLPFC and motor cortex are effective stimulation sites for prevention. When stimulating either one of the two areas, rTMS showed a benefit in the frequency of headache attacks. Some studies have demonstrated that pain was relieved after stimulation in LDLPFC or the motor cortex. However, a combined estimate of effect size indicates that when the LDLPFC or motor cortex is stimulated, rTMS could not improve the pain intensity of chronic migraine.

Based on the results of this study and previous evidence, rTMS could be a beneficial approach to the prevention of migraine re-attack, and stimulation of the left prefrontal cortex was more effective than the motor area. One of the possible reasons for the effectiveness could be modulation of the pain regulation pathway. Previous study suggested that the etiology of migraine is dysfunction of the pain regulation pathway (36), the PET study by Lorenz et al. (37) found that substantial prefrontal cortex activation during heat stimuli on capsaicin-treated skin. Meanwhile, the current fMRI study by Mungoven et al. (38) found that reduced whole scan the dorsolateral prefrontal cortex (DLPFC) connectivity with cortical/subcortical and brainstem regions involved in pain modulation was demonstrated in migraineurs. Furthermore, the functional connection between brain regions that play an important role in regulating pain is significantly weakened, suggesting that migraine could be relieved if the dysfunction of the pain regulation pathway is improved (39). A systematic review showed that areas associated with pain networks can be activated when stimulated by trauma (40), and the DLPFC was believed to be the inhibitory control in pain pathways (41). Therefore, as migraine patients tend to show hyperexcitability of brain cells or cortical dilatation inhibition, rTMS could improve cortical excitability by stimulating the DLPFC and helping to regulate disordered pain neural network connections to prevent migraine.

Previous study has described the effects of rTMS on neurotransmitter systems in rodent (42), but these effects for human being has not been determined until now. In addition to the regulation of the center neural system, another reason by which rTMS improves migraine may be to increase the level of β endorphin (BE) in plasma when stimulated DLPFC. A study revealed that the plasma BE levels of patients with chronic migraine were lower than those in the control group. Three sessions rTMS treatments resulted in remission of migraine and increased plasma BE levels, suggesting that the improved migraine symptoms after rTMS stimulation were associated with increased BE levels (43). However, one of the articles included in this meta-analysis showed that rTMS had no significant effect on improving the frequency of headaches (32). This may be related to the non-double-blinded trial design and the existence of a strong placebo effect. Thus, both the real and sham stimulation groups demonstrated an improvement of the level of BE to reduce the severity and frequency of headaches. So, these results suggested that DLPFC was a key center of pain regulation which may serve as a therapeutic target for migraine.

Another factor that determines the effectiveness of rTMS may depend on the frequency. In this review, high frequency stimulation (≥5 Hz) was used in major studies, except for the study by Teepker et al. (28). In general, high frequency stimulation increases cortical excitability, while low frequency stimulation decreases it (44). This effect seems to contradict with the hyperexcitability of the cortex in migraine patients. Of note, the excitability induced by high-frequency rTMS may be the result of the weakened intracortical or neural network connection inhibition mediated by the gamma-aminobutyric acid (GABA) rather than directly caused by increased excitability (45). The underlying pathophysiological factor of migraine may be low cortical excitability, rather than high excitability (28). From this perspective, high-frequency stimulation may be a better choice for migraine prevention.

rTMS can be used as a preventive treatment for migraine by affecting neurotransmitters and reducing cortical excitability (46, 47). Meanwhile, rTMS stimulation induces synaptic plasticity through long-term enhancement, and repeated stimulation can induce a response for longer than the stimulation period (14). After 5 days of rTMS stimulation, the duration of the strongest analgesic effect is ~1 month, suggesting that repeated stimulation leads to a longer response and obtains a better effect (27).

In total, this meta-analysis adopted the Cochrane systematic review method for research, it provides a direction for future research and clinical treatment. According to this meta-analysis, we preliminary believe that rTMS is of great significance in the prevention of migraine. However, this study still has the following limitations: (1) The efficacy of the rTMS on chronic migraine was preliminary and inconclusive because of the heterogeneity in study designs of rTMS stimulation (including the frequency of stimulation the number of pulse, pulse intensity, and the number of session); (2) The lack of outcomes homogeneity and long-term real world efficacy data, lead to the results do not provide strong evidence to the public; and (3) The sample size is small because of the non-randomized sham-controlled designs, case-reports, had incomplete outcomes, and small sample size (n < 4) were excluded, thus, only eight studies were eligible; (4) As the diagnose criteria used in some studies (25, 28, 33) recruited was ICHD2, the ICHD3 was not adopted in this manuscript, however, from a rigorous perspective, the ICHD third version should be used more in the future study. Finally, none of the eligible trials in this meta-analysis were multicenter trials, and the global reference is therefore limited. So, further high-quality and multicenter trials are needed for confirmation.

## Conclusions

The meta-analysis preliminary revealed that rTMS is an effective approach for reducing migraine re-attack when the DLPFC is stimulated. However, rTMS could not be suggested as a method to reduce the pain level.

## Data availability statement

The original contributions presented in the study are included in the article (or its Supplementary material), further inquiries can be directed to the corresponding authors.

## Author contributions

XH and ZZ designed this study. JZ and ZZ searched literatures, screened the title, and abstracts of studies. YS and YF assessed the quality of the included studies. WL and LY contributed to data analysis. JZ, ZZ, and WL wrote the manuscript. All authors contributed to the article and approved the submitted version.

## Funding

This work was supported by the grant from Basic and Applied Basic Research Fund - Regional Joint Fund of Guangdong Province (Grant No. 2021A1515110162), Characteristic Innovation Project of the Education Department of Guangdong Provincial (Grant No. 2016KTSCX070), and Scientific Research Capacity Improvement Project of Key Construction Disciplines of Guangdong Province (Grant No. 2021ZDJS021).

## Conflict of interest

The authors declare that the research was conducted in the absence of any commercial or financial relationships that could be construed as a potential conflict of interest.

## Publisher's note

All claims expressed in this article are solely those of the authors and do not necessarily represent those of their affiliated organizations, or those of the publisher, the editors and the reviewers. Any product that may be evaluated in this article, or claim that may be made by its manufacturer, is not guaranteed or endorsed by the publisher.

## References

[1] Ashina M Katsarava Z Do TP Buse DC Pozo-Rosich P Özge A . Migraine: epidemiology and systems of care. Lancet. (2021) 397:1485–95. 10.1016/S0140-6736(20)32160-7 33773613

[2] Vos T Abajobir AA Abate KH Abbafati C Abbas KM Abd-Allah F . Global, regional, and national incidence, prevalence, and years lived with disability for 328 diseases and injuries for 195 countries, 1990–2016: a systematic analysis for the Global Burden of Disease Study 2016. Lancet. (2017) 390:1211–59. 10.1016/S0140-6736(17)32154-2 28919117 PMC5605509

[3] Eigenbrodt AK Ashina H Khan S Diener HC Mitsikostas DD Sinclair AJ . Diagnosis and management of migraine in ten steps. Nat Rev Neurol. (2021) 17:501–14. 10.1038/s41582-021-00509-5 34145431 PMC8321897

[4] Marmura MJ Silberstein SD Schwedt TJ. The acute treatment of migraine in adults: the American Headache Society evidence assessment of migraine pharmacotherapies. Headache. (2015) 55:3–20. 10.1111/head.12499 25600718

[5] Orr SL. Diet and nutraceutical interventions for headache management: a review of the evidence. Cephalalgia. (2016) 36:1112–33. 10.1177/0333102415590239 26069242

[6] Worthington I Pringsheim T Gawel MJ Gladstone J Cooper P Dilli E . Canadian Headache Society Guideline: acute drug therapy for migraine headache. Can J Neurol Sci. (2013) 40(5 Suppl 3):S1–S80. 10.1017/S0317167100017819 23968886

[7] Nitsche MA Doemkes S Karakose T Antal A Liebetanz D Lang N . Shaping the effects of transcranial direct current stimulation of the human motor cortex. J Neurophysiol. (2007) 97:3109–17. 10.1152/jn.01312.2006 17251360

[8] Mascarella D Matteo E Favoni V Cevoli S. The ultimate guide to the anti-CGRP monoclonal antibodies galaxy. Neurol Sci. (2022) 2022:1-13. 10.1007/s10072-022-06199-1 35725856

[9] Shehata HS Esmail EH Abdelalim A El-Jaafary S Elmazny A Sabbah A . Repetitive transcranial magnetic stimulation versus botulinum toxin injection in chronic migraine prophylaxis: a pilot randomized trial. J Pain Res. (2016) 9:771–7. 10.2147/JPR.S116671 27785091 PMC5063492

[10] Di Iorio R Rossi S Rossini PM. One century of healing currents into the brain from the scalp: From electroconvulsive therapy to repetitive transcranial magnetic stimulation for neuropsychiatric disorders. Clin Neurophysiol. (2022) 133:145–51. 10.1016/j.clinph.2021.10.014 34864511

[11] Rossi S Antal A Bestmann S Bikson M Brewer C Brockmöller J . Safety and recommendations for TMS use in healthy subjects and patient populations, with updates on training, ethical and regulatory issues: expert guidelines. Clin Neurophysiol. (2021) 132:269–306. 10.1016/j.clinph.2020.10.003 33243615 PMC9094636

[12] Xiong HY Zheng JJ Wang XQ. Non-invasive brain stimulation for chronic pain: state of the art and future directions. Front Mol Neurosci. (2022) 15:888716. 10.3389/fnmol.2022.888716 35694444 PMC9179147

[13] Calabrò RS Billeri L Manuli A Iacono A Naro A. Applications of transcranial magnetic stimulation in migraine: evidence from a scoping review. J Integr Neurosci. (2022) 21:110. 10.31083/j.jin2104110 35864762

[14] Lipton RB Pearlman SH. Transcranial magnetic simulation in the treatment of migraine. Neurotherapeutics. (2010) 7:204–12. 10.1016/j.nurt.2010.03.002 20430320 PMC5084102

[15] Dodick DW Schembri CT Helmuth M Aurora SK. Transcranial magnetic stimulation for migraine: a safety review. Headache. (2010) 50:1153–63. 10.1111/j.1526-4610.2010.01697.x 20553334

[16] Anand S Hotson J. Transcranial magnetic stimulation: neurophysiological applications and safety. Brain Cogn. (2002) 50:366–86. 10.1016/S0278-2626(02)00512-2 12480484

[17] Ridding MC Rothwell JC. Is there a future for therapeutic use of transcranial magnetic stimulation? Nat Rev Neurosci. (2007) 8:559–67. 10.1038/nrn2169 17565358

[18] Charles A. The pathophysiology of migraine: implications for clinical management. Lancet Neurol. (2018) 17:174–82. 10.1016/S1474-4422(17)30435-0 29229375

[19] Gersner R Kravetz E Feil J Pell G Zangen A. Long-term effects of repetitive transcranial magnetic stimulation on markers for neuroplasticity: differential outcomes in anesthetized and awake animals. J Neurosci. (2011) 31:7521–6. 10.1523/JNEUROSCI.6751-10.2011 21593336 PMC6622610

[20] Maeda F Keenan JP Tormos JM Topka H Pascual-Leone A. Modulation of corticospinal excitability by repetitive transcranial magnetic stimulation. Clin Neurophysiol. (2000) 111:800–5. 10.1016/S1388-2457(99)00323-5 10802449

[21] Chen WT Wang SJ Fuh JL Lin CP Ko YC Lin YY. Perictal normalization of visual cortex excitability in migraine: an MEG study. Cephalalgia. (2009) 29:1202–11. 10.1111/j.1468-2982.2009.01857.x 19558536

[22] Feng Y Zhang B Zhang J Yin Y. Effects of non-invasive brain stimulation on headache intensity and frequency of headache attacks in patients with migraine: a systematic review and meta-analysis. Headache. (2019) 59:1436–47. 10.1111/head.13645 31535368

[23] Lan L Zhang X Li X Rong X Peng Y. The efficacy of transcranial magnetic stimulation on migraine: a meta-analysis of randomized controlled trails. J Headache Pain. (2017) 18:86. 10.1186/s10194-017-0792-4 28831756 PMC5567575

[24] Shirahige L Melo L Nogueira F Rocha S Monte-Silva K. Efficacy of noninvasive brain stimulation on pain control in migraine patients: a systematic review and meta-analysis. Headache. (2016) 56:1565–96. 10.1111/head.12981 27869996

[25] Brighina F Piazza A Vitello G Aloisio A Palermo A Daniele O . rTMS of the prefrontal cortex in the treatment of chronic migraine: a pilot study. J Neurol Sci. (2004) 227:67–71. 10.1016/j.jns.2004.08.008 15546593

[26] Fierro B De Tommaso M Giglia F Giglia G Palermo A Brighina F. Repetitive transcranial magnetic stimulation (rTMS) of the dorsolateral prefrontal cortex (DLPFC) during capsaicin-induced pain: modulatory effects on motor cortex excitability. Exp Brain Res. (2010) 203:31–8. 10.1007/s00221-010-2206-6 20232062

[27] Todorov V Bogdanova D Tonchev P Milanov I. Repetitive transcranial magnetic stimulation over two target areas, sham stimulation and topiramate in the treatment of chronic migraine. Comptes Rendus Acad Bulgare Des Sci. (2020) 73:1298–305. 10.7546/CRABS.2020.09.15

[28] Teepker M Hötzel J Timmesfeld N Reis J Mylius V Haag A . Low-frequency rTMS of the vertex in the prophylactic treatment of migraine. Cephalalgia. (2010) 30:137–44. 10.1111/j.1468-2982.2009.01911.x 19515124

[29] Amin R Emara T Ashour S Hemeda M Salah Eldin N Hamed S . The role of left prefrontal transcranial magnetic stimulation in episodic migraine prophylaxis. Egypt J Neurol Psychiatry Neurosurg. (2020) 56:1–6. 10.1186/s41983-019-0140-5

[30] Kumar A Mattoo B Bhatia R Kumaran S Bhatia R. Neuronavigation based 10 sessions of repetitive transcranial magnetic stimulation therapy in chronic migraine: an exploratory study. Neurol Sci. (2020) 42:131–9. 10.1007/s10072-020-04505-3 32556749

[31] Headache Classification Subcommittee of the International Headache Society. The International Classification of Headache Disorders: 2nd edition. Cephalalgia. (2004) 24(Suppl 1):9–160. 10.1111/j.1468-2982.2003.00824.x 14979299

[32] Misra UK Kalita J Tripathi G Bhoi SK. Role of β endorphin in pain relief following high rate repetitive transcranial magnetic stimulation in migraine. Brain Stimul. (2017) 10:618–23. 10.1016/j.brs.2017.02.006 28274721

[33] Misra UK Kalita J Bhoi SK. High-rate repetitive transcranial magnetic stimulation in migraine prophylaxis: a randomized, placebo-controlled study. J Neurol. (2013) 260:2793–801. 10.1007/s00415-013-7072-2 23963471

[34] Sahu A Sinha V Goyal N. Effect of adjunctive intermittent theta-burst repetitive transcranial magnetic stimulation as a prophylactic treatment in migraine patients: a double-blind sham-controlled study. Indian J Psychiatry. (2019) 61:139–45. 10.4103/psychiatry.IndianJPsychiatry_472_18 30992607 PMC6425789

[35] Brighina F Palermo A Daniele O Aloisio A Fierro B. High-frequency transcranial magnetic stimulation on motor cortex of patients affected by migraine with aura: a way to restore normal cortical excitability? Cephalalgia. (2010) 30:46–52. 10.1111/j.1468-2982.2009.01870.x 19438928

[36] Mainero C Boshyan J Hadjikhani N. Altered functional magnetic resonance imaging resting-state connectivity in periaqueductal gray networks in migraine. Ann Neurol. (2011) 70:838–45. 10.1002/ana.22537 22162064 PMC3243965

[37] Lorenz J Cross DJ Minoshima S Morrow TJ Paulson PE Casey KL . unique representation of heat allodynia in the human brain. Neuron. (2002) 35:383–93. 10.1016/S0896-6273(02)00767-5 12160755

[38] Mungoven TJ Marciszewski KK Macefield VG Macey PM Henderson LA Meylakh N. Alterations in pain processing circuitries in episodic migraine. J Headache Pain. (2022) 23:9. 10.1186/s10194-021-01381-w 35033014 PMC8903545

[39] Lim M Jassar H Kim DJ Nascimento TD DaSilva AF. Differential alteration of fMRI signal variability in the ascending trigeminal somatosensory and pain modulatory pathways in migraine. J Headache Pain. (2021) 22:4. 10.1186/s10194-020-01210-6 33413090 PMC7791681

[40] Price DD. Psychological and neural mechanisms of the affective dimension of pain. Science. (2000) 288:1769–72. 10.1126/science.288.5472.1769 10846154

[41] Lorenz J Minoshima S Casey KL. Keeping pain out of mind: the role of the dorsolateral prefrontal cortex in pain modulation. Brain. (2003) 126(Pt 5):1079–91. 10.1093/brain/awg102 12690048

[42] Wassermann EM Lisanby SH. Therapeutic application of repetitive transcranial magnetic stimulation: a review. Clin Neurophysiol. (2001) 112:1367–77. 10.1016/S1388-2457(01)00585-5 11459676

[43] Misra UK Kalita J Tripathi GM Bhoi SK. Is β endorphin related to migraine headache and its relief? Cephalalgia. (2013) 33:316–22. 10.1177/0333102412473372 23314782

[44] Hoogendam JM Ramakers GM Di Lazzaro V. Physiology of repetitive transcranial magnetic stimulation of the human brain. Brain Stimul. (2010) 3:95–118. 10.1016/j.brs.2009.10.005 20633438

[45] Ziemann U TMS. induced plasticity in human cortex. Rev Neurosci. (2004) 15:253–66. 10.1515/REVNEURO.2004.15.4.253 15526550

[46] Keck ME Welt T Müller MB Erhardt A Ohl F Toschi N . Repetitive transcranial magnetic stimulation increases the release of dopamine in the mesolimbic and mesostriatal system. Neuropharmacology. (2002) 43:101–9. 10.1016/S0028-3908(02)00069-2 12213264

[47] Strafella AP Paus T Barrett J Dagher A. Repetitive transcranial magnetic stimulation of the human prefrontal cortex induces dopamine release in the caudate nucleus. J Neurosci. (2001) 21:Rc157. 10.1523/JNEUROSCI.21-15-j0003.2001 11459878 PMC6762641

