# Supplementary material for: Efficacy of repetitive transcranial magnetic stimulation on chronic migraine: A meta-analysis
Source: Front Neurol. 2022 Nov 24;13:1050090. doi: 10.3389/fneur.2022.1050090 (PMC9730425; doi:10.3389/fneur.2022.1050090)
Supplement: Supplementary file 1 [file Data_Sheet_1.pdf]

Strategy of literatures research

Pubmed

("transcranial magnetic stimulation"[MeSH Terms] OR "repetitive transcranial stimulation"[Title/Abstract] OR "rTMS"[Title/Abstract]) AND ("migraine disorders"[MeSH Terms] OR "migraine"[Title/Abstract] OR "migrain*"[Title/Abstract])

Embase

('migraine'/exp OR 'familial migraine' OR 'headache, migrainous' OR 'hemicrania' OR 'migraine' OR 'migraine disorders' OR 'status hemicranicus') AND (rtms OR 'repetitive transcranial magnetic stimulation'/exp OR 'repetitive transcranial magnetic stimulation' OR 'transcranial magnetic stimulation, repetitive')

Embase

('migraine'/exp OR 'familial migraine' OR 'headache, migrainous' OR 'hemicrania' OR 'migraine' OR 'migraine disorders' OR 'status hemicranicus') AND (rtms OR 'repetitive transcranial magnetic stimulation'/exp OR 'repetitive transcranial magnetic stimulation' OR 'transcranial magnetic stimulation, repetitive')

Cochrane

#1: Migraine disorders: Mesh

#2: rTMS: ti, ab, kw OR repetitive transcranial magnetic stimulation: ti, ab, kw OR tms: ti, ab, kw

#3: #1 and #2

Web of science

(ts=tms or ti="repetitive transcranial magnetic stimulation") and (ts=migraine disorder or ti=migrain*)
